# Expression of STEAP1 and STEAP1B in prostate cell lines, and the putative regulation of STEAP1 by post-transcriptional and post-translational mechanisms

**DOI:** 10.18632/genesandcancer.13

**Published:** 2014-03

**Authors:** Inês M. Gomes, Cecília R. Santos, Cláudio J. Maia

**Affiliations:** ^1^ CICS-UBI- Health Sciences Research Centre, University of Beira Interior, Av. Infante D. Henrique, 6200-506 Covilhã, Portugal

**Keywords:** STEAP1, STEAP1B, Prostate cancer, Post-transcriptional, Post-translational

## Abstract

STEAP1 gene is overexpressed in several kinds of tumors, particularly in prostate cancer. Besides STEAP1, there is another related gene, STEAP1B, which may encode two different transcripts. Although several studies have been pointing STEAP1 as a putative immunotherapeutic target and biomarker, the mechanisms underlying its regulation are not fully understood. *In silico* analysis allowed us to show that STEAP1 and STEAP1B share high homology, but with slight differences at structural level. Experiments with prostate cells showed that STEAP1B2 is overexpressed in cancer cells. Regarding STEAP1 regulation, it is demonstrated that the stability of mRNA and protein is higher in LNCaP than in PNT1A cells. Of note, serum triggered opposite effects in LNCaP and PNT1A in relation to STEAP1 stability, e.g., increasing it in PNT1A and decreasing in LNCaP. These results suggest that STEAP1 may be regulated by post-transcriptional and post-translational modifications (PTM), which may differ between non-neoplastic and neoplastic cells. These PTM are supported through *in silico* analysis, where several modifications such as N-glycosylation, N-Glycation, Phosphorylation and O-linked β-N-acetylglucosamine, may occur in STEAP1 protein. In conclusion, these data indicate that STEAP1B2 is overexpressed in neoplastic cells, and PTM may be involved in regulation of STEAP1 expression in prostate cells.

## INTRODUCTION

The six transmembrane epithelial antigen of the prostate 1 (STEAP1) is overexpressed in prostate cancer, and other malignant tumors with slightly less intensity [1-4]. Regarding normal tissues, STEAP1 expression is almost restricted to prostatic cells preferentially located on the plasma membrane of epithelial cells, particularly on cell-cell junctions, and to a lesser extend dispersed on the cytoplasm [2, 3, 5]. Although its precise cellular function is still not fully understood, STEAP1 appears to function as a transporter or ion channel, taking part on inter- and intracellular communication, possibly regulating cell proliferation and invasiveness [1-3, 6]. Several studies have been pointed out the STEAP1 as a potential immunotherapeutic target as well as a biomarker, emphasizing its clinical relevance [7-11]. Besides STEAP1 gene, another related gene, STEAP1B, is encoded by the human genome. This gene is located at a different arm of the same chromosome as STEAP1, and may originate two different transcripts, STEAP1B1 and STEAP1B2. However, its expression in prostate cells is unstudied. Recently, our research group has demonstrated that STEAP1 is regulated by androgens and estrogens on the LNCaP cell line [5]. However, the mechanisms underlying STEAP1 over-expression on prostate cancer remain to be elucidated. It is well known that the regulation of gene expression could occur not only at transcription level but also at post-transcriptional and post-translational levels. In fact, events like cell-cycle progression, signal transduction and apoptosis, which are closely associated with oncogenesis, are influenced by mRNA stability as well as the rate of protein degradation [12-17]. It has been reported that tumors may activate endogenous mechanisms to increase mRNA stability of genes encoding oncogenes, cytokines, growth factors, and enzymes, leading to an enhanced protein over-expression, consequently raising cell growth and inflammatory processes known to be involved in the onset and progression of carcinogenesis [18-23]. Post-translational modifications (PTM) are intrinsically involved on regulating protein function, and therefore, are crucial for a variety of cellular processes, such as transcription, replication, cell cycle, apoptosis and cell signaling [24, 25]. Overall, we aimed to conduct an extensive *in silico* analysis of STEAP1 and STEAP1B, and to evaluate STEAP1 and STEAP1B expression in human prostate cell lines. In addition, the putative post-transcriptional and PTM modifications are evaluated through STEAP1 mRNA and protein stability, supplemented by a post-translational *in silico* analysis.

## RESULTS

### STEAP1 and STEAP1B gene share high homology and are differentially expressed in human prostate cell lines

**Figure 1:**
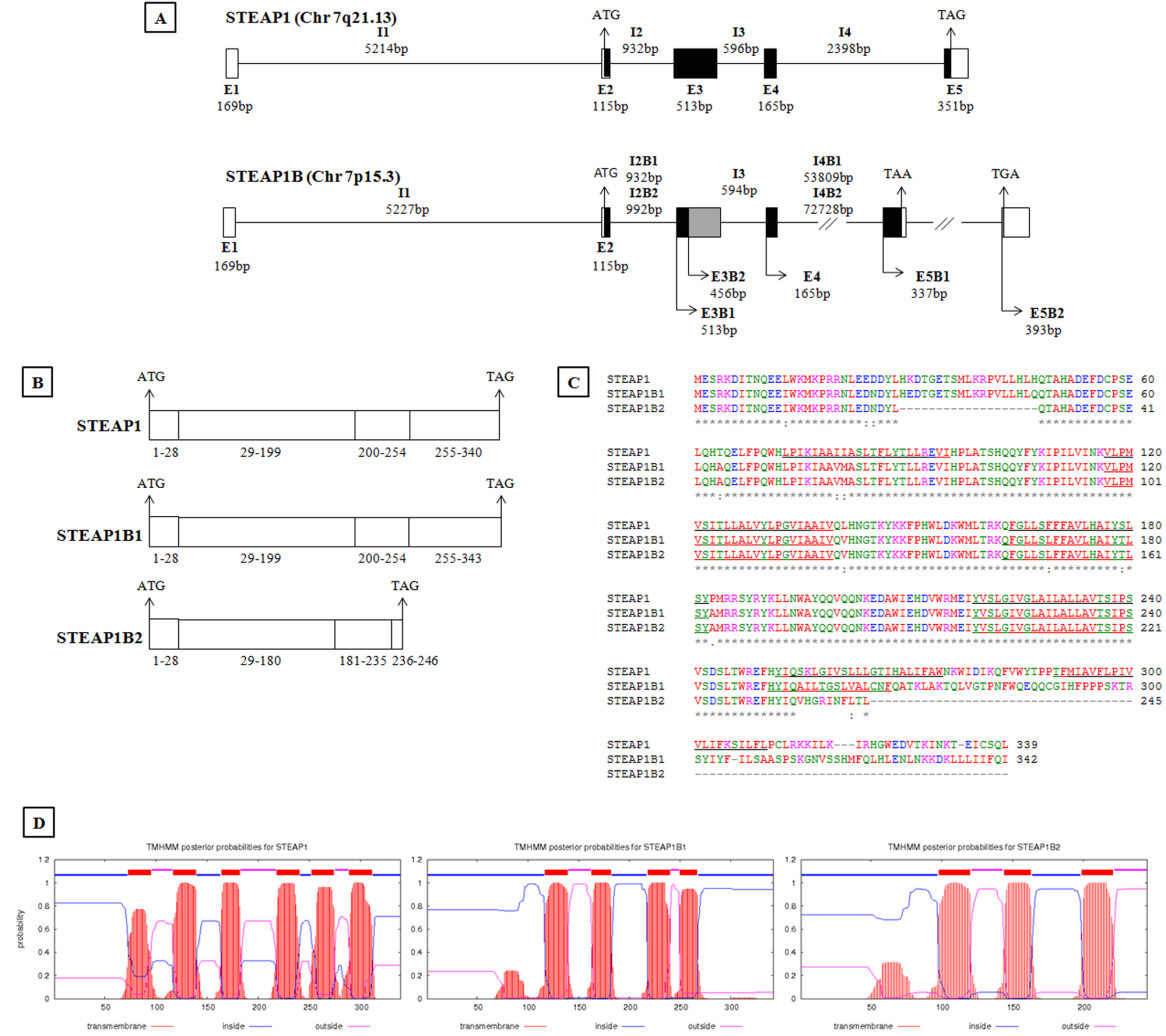
In silico analysis of human STEAP1 and STEAP1B gene Genomic organization (A) and transcripts (B) resulting from STEAP1 and STEAP1B gene. Exons (E), Introns (I) and their molecular sizes in bp (base pairs) are indicated. The sequence ATG and TAG/TAA corresponds to initiation and STOP codons, respectively. White boxes indicate non-coding exons, and black or grey boxes represent regions of coding exons depending on transcript encoded by STEAP1B gene. C- Alignment of amino acids sequences of STEAP1 and putative STEAP1B isoforms. The underlined amino acids sequences correspond to predicted transmembrane regions. * indicate identical amino acids among STEAP1s proteins; “:” indicate different amino acids but with similar physical-chemistry properties. D- Prediction of transmembrane helices in STEAP1, STEAP1B1 and STEAP1B2 proteins. All sequences were retrieved from http://genome.ucsc.edu/ and the alignment was carried out using Clustal Omega (https://www.ebi.ac.uk/Tools/msa/clustalo/). The prediction of transmembrane helices was performed resorting to Center for Biological Sequence analysis (http://www.cbs.dtu.dk/services/TMHMM/).

A detailed *in silico* analysis allowed to compare the genomic organization of STEAP1 and STEAP1B genes. STEAP1 gene is found close to the telomeric region on chromosome 7q21.13, encoding a transcript with 1.3 Kb, which originates a 339 aa mature protein (39.72KDa), with six predicted transmembranar regions, connected by three extracellular and two intracellular loops, and both COOH and NH_2_ intracellular terminal. The transmembrane domains are thought to be located between 73-95, 117-139, 164-182, 218-240, 252-274, 289-311 of the aa sequence (www.ncbi.nlm.nih.gov/protein/NP_036581.1; http://www.cbs.dtu.dk/services/TMHMM/). STEAP1B gene is localized on chromosome 7p15.3 and may originate two different transcripts, namely STEAP1B1 and STEAP1B2 (Figure 1). In comparison to the STEAP1 gene, STEAP1B1 has an additional exon, and a very large intron 4, with 53809bp on STEAP1B1 and 72728bp on STEAP1B2. STEAP1B1 is the longer transcript spanning approximately 1.3 Kb, and may encode the longer isoform with 342 aa (39.547KDa), containing four potential transmembranar regions between aa 117-139, 163-182, 218-240 and 250-267, two intracellular and two extracellular loops, and COOH and NH_2_ intracellular terminal regions (www.ncbi.nlm.nih.gov/protein/NP_001157932.1; http://www.cbs.dtu.dk/services/TMHMM/). STEAP1B2 transcript has approximately 1.2Kb, and may encode a protein with 245 aa (28.684 KDa) with three potential transmembranar regions situated between aa 98-120, 144-163 and 199-221, one intracellular and one extracellular loops, an intracellular NH_2_ termini and an extracellular COOH region (www.ncbi.nlm.nih.gov/protein/NP_997225.1; http://www.cbs.dtu.dk/services/TMHMM/) (Figure 1). STEAP1B2 uses an alternate in-frame splice site in the 5' coding region and an alternate 3' exon with a distinct 3' coding region and 3' UTR, compared to variant 1. The resulting isoform lacks an internal segment near the N-terminus and has a shorter and distinct C-terminus when compared to isoform 1 (http://www.ncbi.nlm.nih.gov/gene?term=STEAP1B). This detailed analysis reveals that STEAP1 and STEAP1B1 isoforms share 89% and STEAP1 and STEAP1B2 91% of homology (http://blast.ncbi.nlm.nih.gov/Blast.cgi).

**Figure 2:**
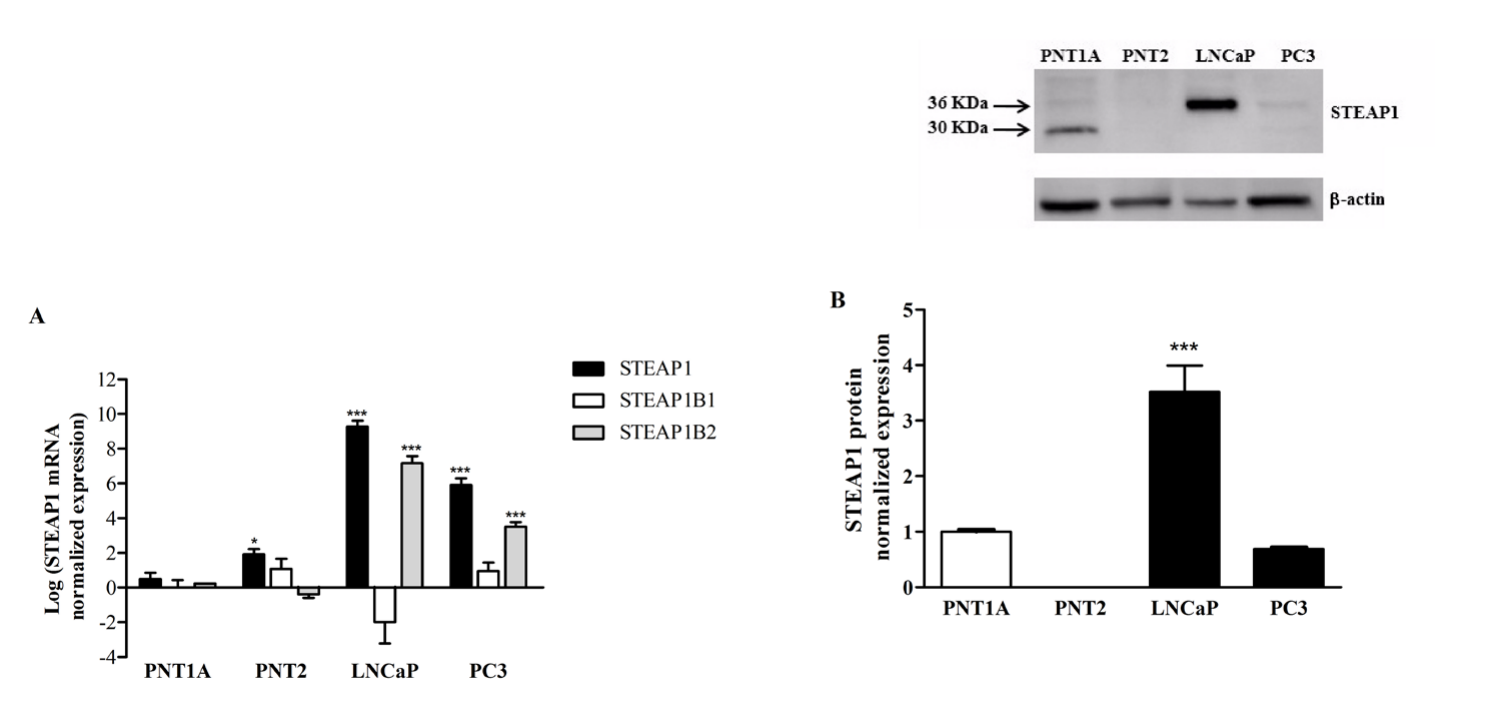
Differential expression of STEAP1, STEAP1B1 and STEAP1B2 on prostate cell lines A. mRNA expression of STEAP1 and its isoforms were determined by qPCR. B. STEAP1 protein expression was determined by Western blot. mRNA and protein expression was normalized with hGAPDH and β-actin, respectively. Error bars indicate mean ± SEM of n=6. *p<0.05, ***p<0.001 (One-way ANOVA followed by Bonferroni test) compared with PNT1A expression.

STEAP1, STEAP1B1 and STEAP1B2 mRNA are differentially expressed in prostate cells lines. On the non-neoplastic prostate cells, PNT1A and PNT2, STEAP1, STEAP1B1 and STEAP1B2 mRNAs have little to no expression. On the other hand, on the malignant prostate cells, LNCaP and PC3, STEAP1 and STEAP1B2 are highly expressed, particularly STEAP1 (Figure 2A). STEAP1B1 mRNA is mainly expressed on PNT2 and PC3 cells, and under-expressed on LNCaP cells. The expression of STEAP1 protein was evaluated by western blot analysis (Figure 2B). Two immunoreactive bands could be identified, one of 30 KDa on PNT1A and other of 36 KDa on LNCaP cells. As seen in Figure 2B, STEAP1 protein is highly expressed on LNCaP cells, followed by PNT1A, PC3 and PNT2 with no expression.

### Stability of STEAP1 mRNA and protein in human prostate cell lines

**Figure 3:**
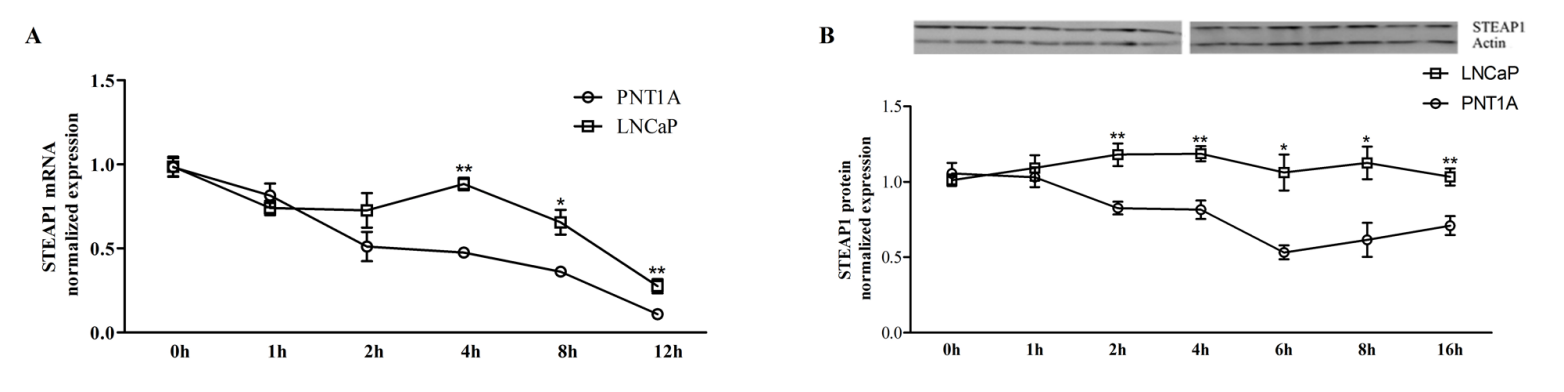
STEAP1 mRNA and protein stability in human prostate cell lines Comparison between PNT1A and LNCaP STEAP1 mRNA (A) and protein (B) stability in complete culture medium. mRNA and protein expressions were determined by qPCR and Western blot, respectively. mRNA and protein expression was normalized with hGAPDH and β-actin, respectively. Error bars indicate mean ± SEM of n=6. *p<0.05, p**<0.01 (t-test) comparing PNT1A and LNCaP STEAP1 expression at each indicated time.

To evaluate the hypothesis that post-transcriptional and PTM mechanisms could be involved in the differential expression of STEAP1 between non-neoplastic and neoplastic cells, PNT1A and LNCaP cells were chosen to determine STEAP1 mRNA and protein stability through qPCR and western blot analysis, respectively. Cells were primarily cultured on CM, and STEAP1 mRNA stability was established by assessing the relative decay rates of STEAP1 after Act D treatment at 0, 1, 2, 4, 8 and 12h (Figure 3A). STEAP1 protein stability was determined after inhibiting protein synthesis with Chx for 0, 1, 2, 4, 6, 8 and 16h (Figure 3B). On PNT1A cells, the approximate half-life of STEAP1 mRNA levels is 4h, whereas on LNCaP is about 10h. Comparing the decaying mRNA levels of both cell lines, it is noticed that STEAP1 mRNA is more stable on LNCaP cells than on PNT1A cells. Regarding to STEAP1 protein expression after exposure to Chx, the approximate half-life of STEAP1 protein on PNT1A cells is about 6h, whereas the levels on LNCaP are still high even after 16h of treatment with Chx. In accordance to the mRNA data, it is clear that STEAP1 protein is also more stable on LNCaP than on PNT1A cells. However, no relationship seems to occur between the decay rates of mRNA and protein, namely on LNCaP cells.

**Figure 4:**
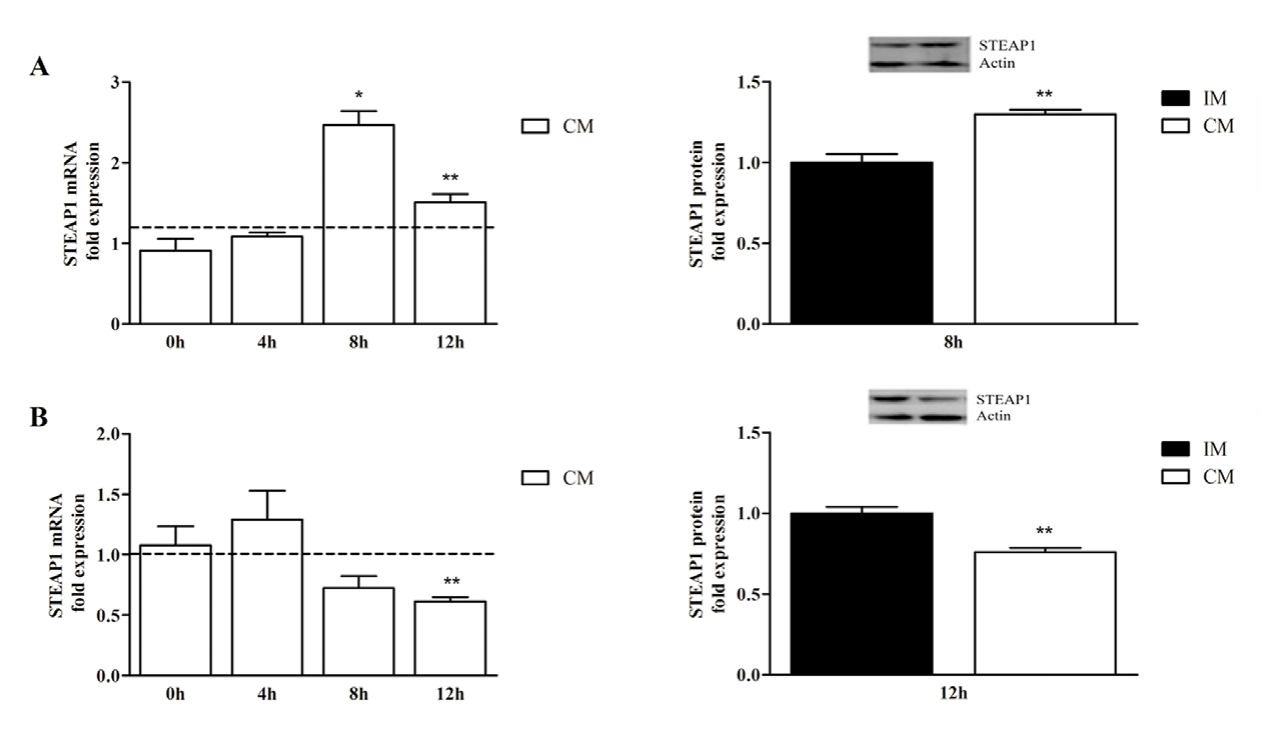
Effect of serum on PNT1A (A- upper left and right) and LNCaP (B- lower left and right) STEAP1 mRNA and protein stability, after treatment with Act D and Chx, respectively mRNA and protein fold variation relatively to incomplete medium (IM) were determined by qPCR and Western blot, respectively. mRNA and protein expression was normalized with hGAPDH and with β-actin, respectively. Error bars indicate mean ± SEM of n=6. *p<0.05, **p<0.01 (t- test) comparing (IM) and complete medium (CM) STEAP1 expression at each indicated time.

It is well known that FBS (serum) contains hormones and growth factors important to cell homeostasis. Therefore, the effect of serum on STEAP1 mRNA and protein stability was investigated. In the presence of FBS, STEAP1 mRNA expression increased 2 fold after 8h of stimuli on PNT1A cells (Figure 4A, upper left), whereas on LNCaP cells STEAP1 mRNA expression decreased almost 2 fold after 12h of stimuli with FBS (Figure 4B, lower left). The effect of FBS in STEAP1 protein levels at these time points was in accordance to STEAP1 mRNA, i.e., increased at 8h on PNT1A (Figure 4A right upper) and decrease after 12h on LNCaP cells (Figure 4B, lower right). These results show a clear opposite response to hormones and growth factors, which are present in FBS, in non-neoplastic and neoplastic prostate cell lines.

### *In silico* analysis of post-translational modifications in STEAP1 protein

**Figure 5:**
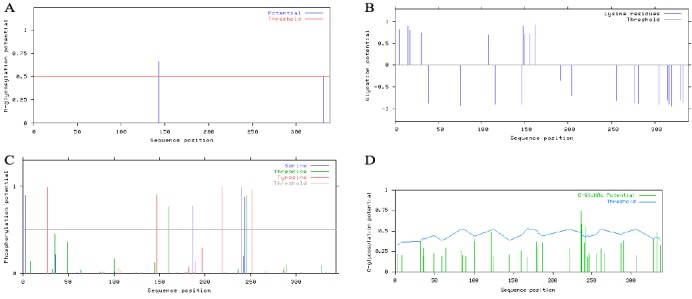
Prediction of N-glycosylation, glycation, phosphorylation and O-β-GlcNAc sites of STEAP1 using: A. NetNGlyc 1.0, B- NetGlycate 1.0, C. NetPhos 2.0 and D- YinOyang 1.2, respectively

In an attempt to uncover the causes behind the above mentioned differences on STEAP1 protein stability, a closer insight into PTM was achieved through an extensive *in silico* analysis. Alterations such as N-glycosylation, glycation, phosphorylation sites and O-β-GlcNAc anchor sites on STEAP1 aa sequences were investigated. As seen on Figure 5A, two N-X-S/T consensus sequences [32] were found on STEAP1 sequence, but a single potential N-glycosylation site was identified at position 143, corresponding to the asparagine aa. Although another asparagine was signaled, the potential score was below the threshold line. Potential glycation sites occur in several lysines (9/24) of STEAP1 protein. These potentially glycated aa are located at positions 5, 15, 17, 30, 108, 148, 149, 156 and 162 (Figure 5B). It is well known that three distinct aa (serine, threonine and tyrosine) have the ability to be phosphorylated. According to the results retrieved from NetPhos 2.0 Server, four serines (positions 3, 187, 240 and 244), two threonines (positions 160 and 246) and four tyrosines (positions 27, 147, 219 and 252) are potentially phosphorylated on STEAP1 (Figure 5C). Several kinase enzymes with the potential to phosphorylate different aa on STEAP1 sequence were also identified. As seen on Table 2, the threonine at position 160 presents the highest score to be phosphorylated by protein kinase C. GlcNAcylation usually occurs in parallel with phosphorylation, and the two are frequently mutually exclusive [33, 34]. In fact, the potential O-β-GlcNAc anchor sites can be found on threonines 236 and 333, as well as on serines 237 and 242 of the STEAP1 aa sequence (Figure 5D).

**Table 1:** Sequences, amplicons sizes and annealing temperatures for the different STEAP1 mRNA and the internal controls used for quantitative Real-Time PCR

Oligo name	Sequence (5ʹ-3ʹ)	Amplicon Size (bp)	Anneling temperature
hSTEAP1_619fw	GGC GAT CCT ACA GAT ACA AGT TGC	128	60°C
hSTEAP1_747rv	CCA ATC CCA CAA TTC CCA GAG AC		
hSTEAP1B1fw	CTGGAAGCCTGGTAGCTTTG	162	
hSTEAP1B1rv	GGCTGGCTGCTGATAAAATG		
hSTEAP1B2fw	CGATTATTTGCAAACAGCCC	173	
hSTEAP1B2rv	GGGAAGTTGCTAAAGGGTGA		
hGAPDH_74fw	CGC CCG CAG CCG ACA CAT C	75	
hGAPDH_149rv	CGC CCA ATA CAA TCC G		

**Table 2:** Putative Kinases enzymes involved on STEAP1 phosphorylation Prediction was performed using NetPhosK 1.0 server.

Site	Kinase	Score
S-3	PKC	0,67
T-8	CKII	0,57
Y-27	EGFR	0,58
Y-27	INSR	0,55
T-64	DNAPK	0,59
T-64	ATM	0,60
S-83	DNAPK	0,55
S-83	PKA	0,74
T-85	PKC	0,64
T-100	PKC	0,67
S-122	PKA	0,67
T-145	PKC	0,59
T-160	PKC	0,91
S-179	PKC	0,59
S-181	PKC	0,68
S-187	PKC	0,87
S-187	PKA	0,58
Y-197	EGFR	0,60
S-221	PKA	0,55
T-246	CKII	0,53
T-246	PKC	0,86
S-255	PKC	0,78
S-255	Cdc2	0,56
S-261	PKA	0,68
T-287	Cdk5	0,52
S-306	PKA	0,63
T-333	PKC	0,63
S-337	DNAPK	0,58
S-337	ATM	0,64

## DISCUSSION

STEAP1 is mainly expressed in normal and malignant prostate and the pursuit for understanding how its expression is regulated and modulated by internal and external factors is fundamental. Recently, Grunewald and colleagues described the existence of a STEAP1 homolog gene, named STEAP1B [35]. STEAP1 and STEAP1B genes are located on chromosome 7, but on different arms, i.e., STEAP1 gene is located at the long arm (7q21.13) and STEAP1B on the short arm (7p15.3). Considering the high homology between STEAP1 and STEAP1B genes, it is possible that gene duplication may have occurred during genome evolution. STEAP1B gene gives rise to two transcripts, STEAP1B1 and STEAP1B2, which share several similarities to STEAP1, but may encode two different mature proteins of 332 aa and 245 aa, respectively. Although the expression of STEAP1B at protein level still needs to be verified, these putative proteins also display transmembrane regions like STEAP1, but in a smaller number, suggesting that different roles could be played by these isoforms in comparison to STEAP1 protein. There are some common features present on the structure of STEAP family of proteins, such as the presence of an N-terminal with homology to the archaeal and bacterial F_420_H_2_:NADP^+^ oxidoreductase (FNO)–binding proteins and human NADPH-oxidoreductases, two conserved histidines that bind an intramembranar heme group known as the ACRATA domain, the presence of the Rossman fold within the N-terminal and the strikingly similarity between the C-terminal domain to the yeast FRE cytochrome b metalloreductase domain, indicating their possible role as oxidoreductases [36-39]. Both STEAP1B isoforms, lack the FNO-like domain, the Rossman fold and the NADPH-oxidoreductase domain, as STEAP1 [35, 36]. In addition, STEAP1B2 appears to be also missing the heme-binding sites, making it unfit to act as a ferric oxidoreductase [35].

STEAP1 mRNA overexpression is well described on LNCaP and PC3 cells [2]. We demonstrated that STEAP1B1 and STEAP1B2 mRNAs are also expressed on prostate cell lines. Alike in STEAP1, STEAP1B2 mRNA is also overexpressed in neoplastic cells when compared to non-neoplastic cells, suggesting that also STEAP1B2 may be dysregulated in cancer and demonstrating its potential application as a biomarker. STEAP1B1 mRNA can only be found in diminished levels on PNT2 and PC3 cells, and little or no expression on the LNCaP and PNT1A cells. These results seem to suggest that STEAP1B1 mRNA is not differentially expressed between normal and prostate cancer. However, it is needed to carry out studies using human prostate cancer cases to evaluate the expression profile of STEAP1B1 and STEAP1B2, as well as to clarify their clinical significance.

A strong immunoreactive band of approximately 36 KDa was detected on LNCaP cells, which must correspond to STEAP1 protein, as described by others [1, 2, 6]. Interestingly, an immunoreactive band of 30 KDa was obtained on PNT1A cells. The different molecular weight of STEAP1 between LNCaP and PNT1A may result from different PTM on protein, as suggested by our results and discussed below. It is also possible that the immunoreactive band of 30 KDa may correspond to STEAP1B2, because this is the predicted molecular weight of STEAP1B2 isoform. Nonetheless, further analysis using specific antibodies against STEAP1B1 and STEAP1B2 are required to determine their expression.

Numerous studies have described that STEAP1 is overexpressed in several kinds of tumors, but the mechanisms underlying its overexpression remain to be clarified. With this work we also attempted to unveil if STEAP1 expression would be regulated by post-transcriptional and PTM. Considering that both mRNA and protein stability display an important role at cellular level, in regulation of gene induction/expression, proliferation, cell signaling and apoptosis [12-16, 40], the STEAP1 stability in non-neoplastic and neoplastic prostate cell lines was evaluated. mRNA half-life and turnover are dependent on development stage or environmental factors, such cytokines, hormones, among others, decreasing its rate of transcription [40, 41]. The regulation of mRNA stability is intrinsically associated to the regulation of protein production [40]. The most stable mRNA and proteins are usually associated with vital processes such as translation, metabolic pathways and respiratory machinery [42]. According to the collected data, STEAP1 mRNA and protein are less stable in the PNT1A cells in comparison to the highly stable STEAP1 mRNA and protein on LNCaP cells. The enhancement of STEAP1 stability in LNCaP cells suggests that post-transcriptional and PTM may differ between non-neoplastic and neoplastic cells, contributing for STEAP1 overexpression in cancer cells. The evaluation of the effect of serum on STEAP1 stability shows that serum has opposite effects on STEAP1 stability, increasing it on PNT1A and decreasing on LNCaP cells. Recently, our research group demonstrated that treatment with serum or DHT down-regulates STEAP1 expression through androgen receptor (AR) [5]. Considering that DHT increases AR mRNA stability and consequently the levels of AR protein, it is liable to speculate that DHT present on serum may contribute to decreased STEAP1 stability [43]. However, other factors may be involved in stability of STEAP1.

The opposite effect of serum in STEAP1 stability on PNT1A cells reinforce that the mechanisms involved in regulation of STEAP1 may differ between non-neoplastic and neoplastic cells. Furthermore, the increased stability of both mRNA and protein on LNCaP cells is in accordance that stable mRNAs allow a wider translational timeframe for genes that are expressed at high levels [44].

There are several factors that could control mRNA and protein turnover on cells, and in an attempt to determine possible PTM's in STEAP1 protein, *in silico* analysis was carried out. Four different types of PTM's were found to be likely to occur in STEAP1 protein, namely N-glycosylation, glycation, phosphorylation and O-β-GlcNAcylation. STEAP1 protein is therefore potentially subjected to multisite modification, a phenomenon that modulates protein function by “loss-of-function” and/or “gain-of-function” mechanisms as a result of interaction between the different modifications [25]. It is well documented that these types of modifications tend to confer higher stability to proteins and are often implicated on the etiology and pathogenesis of several diseases, including cancer [25, 45-50]. Furthermore, certain PTM's may even be used as diagnostic targets and are known to enhance tumor cell proliferation and invasion, as in prostate cancer [51-53]. Further analysis is required to ascertain if these particular PTM's are occurring on cells, and if they occur, it is crucial to evaluate the role of these modifications in carcinogenesis.

In conclusion, STEAP1B transcripts have similar structural features to STEAP1, but may encode proteins with less transmembrane domains. STEAP1B2 transcript is also overexpressed on neoplastic prostate, making it worth to evaluate its potential as cancer biomarker. For the first time, we demonstrated that STEAP1 expression and regulation could also be under the control of several PTM. Clarifying the regulation of STEAP1 as well as the expression and function of STEAP1B on cells may open novel strategies for diagnosis and treatment of prostate cancer.

## MATERIAL AND METHODS

### Cell culture and treatment

Human prostate cell lines (PNT1A, PNT2, LNCaP and PC3) were purchased from the European Collection of Cell Cultures (ECACC, Salisbury, UK). All cell lines were grown in RPMI 1640 medium (Gibco, Paisley, Scotland) supplemented with 10% FBS (Biochrom AG, Berlin, Germany) and 1% penicillin/streptomycin (Invitrogen, NY, USA), in a humidified chamber at 37°C and a 5% CO_2_ atmosphere. Determination of STEAP1, STEAP1B1 and STEAP1B2 expression was determined through the collection of cells from three different passages. Evaluation of mRNA and protein stability was achieved with treatment with inhibitors of transcription [actinomycin D (Act D)], and translation [cycloheximide (Chx)], respectively. Two different approaches were carried out in order to evaluate STEAP1 mRNA and protein stability in cells: a) LNCaP and PNT1A cells were grown up to 60% confluence in Complete Medium [(CM) RPMI 1640, 10% FBS and1% penicillin/streptomycin]. This medium was then replaced by CM supplemented with Act D 1μg/mL, or Chx 1μg/mL. Cells were harvested after 0h, 1h, 2h, 4h, 8h and 12h to assess mRNA stability, and after 0h, 1h, 2h, 4h, 8h and 16h for protein stability; b) LNCaP and PNT1A cells were grown up to 60% in Incomplete Medium [(IM) without serum]. This medium was posteriorly supplanted by CM or IM supplemented with Act D 1μg/mL, or Chx 1μg/mL. Cells were harvested after 0h, 4h, 8h and 12h for mRNA stability, and after 8h and 12h for protein stability in PNT1A and LNCaP cells, respectively.

### Total RNA extraction and cDNA synthesis

Total RNA from human prostate cell lines was obtained using TRI reagent (Ambion, UK) according to the manufacturer's instructions. Total RNA integrity and quantification were assessed by agarose gel electrophoresis and measuring 260 and 280ηm absorvances on a nanospectrometer (Pharmacia Biotech, Ultraspec 3000). cDNA synthesis was carried out using NZY First-Strand cDNA Synthesis Kit (Nzytech, Lisboa, Portugal) according to protocol.

### Total Protein Extraction

PNT1A, PNT2, LNCaP and PC3 cells were lysed on an appropriated volume of RIPA buffer (150 mM NaCl, 1% Nonidet-P40 substitute, 0.5% Na-deoxycholate, 0.1% SDS, 50 mM Tris, 1 mM EDTA) supplemented with 1% protease cocktail and 10% PMSF. The total protein extract (supernatant) was obtained after centrifugation the cell lysate for 20min at 12000 rpm and 4°C. Quantification of the total protein bulk was measured using the Bradford method (Biorad, CA, USA).

### Quantitative Real-Time PCR analysis

Quantitative real-time PCR (qPCR) was used to determine the expression of STEAP1, STEAP1B1 and STEAP1B2 in prostate cell lines and to establish PNT1A and LNCaP half-life of STEAP1 mRNA. qPCR reactions were performed on IQ5 Multicolor qPCR Detection System (Bio-Rad, Hercules, USA) using Maxima™ SYBR Green/Fluorescein qPCR Master Mix (Thermo Scientific, Vilnius, Lithuania). The efficiency of qPCR was determined for all designated primers (Table 1) with serial dilutions (1:1; 1:10; 1:100; 1:1,000) of the cDNA. qPCR reactions were performed using 1μl of cDNA in a 20μl reaction containing 10μl SYBR Green and 300nM of specific primers. After an initial denaturation at 95 °C for 5min, 35 cycles were carried out as follows: denaturation at 95 °C for 30s, annealing temperature for 30s and polymerization at 72 °C for 20s. The amplified PCR fragments were analyzed by melting curves: reactions were heated from 55 to 95°C with 10s holds at each temperature (0.05°C/s). Fold differences were calculated following the mathematical model proposed by Pfaffl [26].

### Western blot

Approximately 80μg of total protein from PNT1A, PNT2, PC3 and LNCaP cells were used to assess the STEAP1 protein expression on the four cell lines, and to determine protein stability. The protocol followed as previously been described by us [27]. Briefly, LNCaP and PNT1A protein extracts were resolved on 12% SDS-PAGE electrophoresis gel and then transferred into a PVDF membrane (GE Healthecare, UK). After blockage with 3% casein solution, membranes were incubated with STEAP1 rabbit polyclonal antibody against humanSTEAP1 (H105) diluted 1:300 (Santa Cruz Biotechnology, Santa Cruz). Membranes were then incubated with an alkaline phosphatase conjugated goat polyclonal antibody against rabbit IgG (GE Healthcare, UK). Finally, STEAP1 immunoreactivity was visualized using Molecular Imager FX (Biorad, Hercules) after a brief incubation with ECF substract. STEAP1 and β-actin expression levels were quantified by densitometry using Quantity One Software (Biorad). β-actin was used to normalize STEAP1 expression.

### Bioinformatic analysis

mRNA and protein sequences of STEAP1, STEAP1B1 and STEAP1B2 were retrieved from the National Center for Biotechnology Information (http://www.ncbi.nlm.nih.gov/). Alignment of amino acid (aa) sequences was performed using Clustal Omega program (https://www.ebi.ac.uk/Tools/msa/clustalo/). To determine protein homology, the online Basic Local Alignment Search Tool program (BLAST) (http://blast.ncbi.nlm.nih.gov/Blast.cgi) was used. Prediction of putative transmembranar domains was determined using TMHMM Server v. 2.0 (http://www.cbs.dtu.dk/services/TMHMM). Prediction of amino acid post-translational alterations such as N-glycosylation, N-Glycation, Phosphorylation and Phosphokinase specific sites, and addition sites of O-linked β-N-acetylglucosamine (O-β-GlcNAc) were carried out using several tools available on Expasy (http://www.expasy.org/proteomics/post-translational_modification), NetNGlyc 1.0, NetGlycate 1.0 [28], NetPhos 2.0 [29], NetPhosK 1.0 [30] and YinOYang 1.2 [31] software's, respectively.

### Statistical analysis

Data from all experiences are shown as mean ± SEM of n=6. The statistical significance of STEAP1, STEAP1B1 and STEAP1B2 was assessed using One-way ANOVA followed by Bonferroni test. mRNA and protein stability experiments statistical data was obtained comparing PNT1A and LNCaP STEAP1 expression at each indicated time, using t-test.
